# Digital Microfluidic qPCR Cartridge for SARS-CoV-2 Detection

**DOI:** 10.3390/mi13020196

**Published:** 2022-01-27

**Authors:** Kuan-Lun Ho, Hong-Yu Liao, Helene Minyi Liu, Yen-Wen Lu, Pin-Kuan Yeh, Justin Yu Chang, Shih-Kang Fan

**Affiliations:** 1 Department of Mechanical and Nuclear Engineering, Kansas State University, Manhattan, KS 66506, USA; kuanlun@ksu.edu; 2 Department of Mechanical Engineering, National Taiwan University, Taipei 10617, Taiwan; r07522320@ntu.edu.tw; 3 Graduate Institute of Biochemistry and Molecular Biology, National Taiwan University, Taipei 10052, Taiwan; mliu@ntu.edu.tw; 4 Department of Bio-Industrial Mechatronics Engineering, National Taiwan University, Taipei 10617, Taiwan; yenwenlu@ntu.edu.tw; 5 GEMFluidix LLC, Los Angeles, CA 91748, USA; vincentyeh@gemfluidix.com (P.-K.Y.); justinchang@gemfluidix.com (J.Y.C.)

**Keywords:** SARS-CoV-2, COVID-19, droplet qPCR, digital microfluidics, electrowetting

## Abstract

Point-of-care (POC) tests capable of individual health monitoring, transmission reduction, and contact tracing are especially important in a pandemic such as the coronavirus disease 2019 (COVID-19). We develop a disposable POC cartridge that can be mass produced to detect the SARS-CoV-2 N gene through real-time quantitative polymerase chain reaction (qPCR) based on digital microfluidics (DMF). Several critical parameters are studied and improved, including droplet volume consistency, temperature uniformity, and fluorescence intensity linearity on the designed DMF cartridge. The qPCR results showed high accuracy and efficiency for two primer-probe sets of N1 and N2 target regions of the SARS-CoV-2 N gene on the DMF cartridge. Having multiple droplet tracks for qPCR, the presented DMF cartridge can perform multiple tests and controls at once.

## 1. Introduction

As the pandemic of coronavirus disease 2019 (COVID-19) caused by severe acute respiratory syndrome coronavirus 2 (SARS-CoV-2) is ongoing, there is an urgent demand for low-cost, fast, sensitive, and reliable assays to support the diagnosis and management of SARS-CoV-2 infection [1]. The SARS-CoV-2 virus belongs to the *Coronaviridae* family, which contains a single positive strand of RNA that encodes spike glycoprotein (S), envelope protein (E), membrane protein (M), and nucleoprotein (N) [2]. Although effective vaccines against COVID-19 are available, healthy people still have a high probability of infection due to the presence of highly transmissible variants and a large number of infected individuals worldwide [3]. Therefore, early and rapid detection is particularly important in the prevention of further transmission to control the COVID-19 pandemic.

Other than detecting anti-SARS-CoV-2 immunoglobulin M (IgM) and immunoglobulin G (IgG) in blood samples [4,5], the most common COVID-19 viral tests [6,7,8] are: nucleic acid amplification tests (NAATs) of viral RNA genes [9,10] and immunoassays of viral antigens [11,12] with nasopharyngeal or oropharyngeal specimens. In general, antigen tests are rapid and less expensive, whereas NAATs are considered as the “gold standard” for clinical diagnostic detection holding high sensitivity and specificity but are typically processed with a long turnaround time. Due to the high mutation frequency in the spike glycoprotein coding region [13], the detection of nucleoprotein coding sequences, which is highly conserved among SARS-CoV-2 isolates, is more feasible than that of the spike glycoproteins or other coding sequences [14,15]. In addition, the nucleoproteins of many coronaviruses are highly immunogenic and are expressed in large quantities during the infection period [15], thus it is targeted by the Centers for Disease Control and Prevention (CDC, USA) reverse transcription real-time quantitative polymerase chain reaction (RT-qPCR) panel for the detection of SARS-CoV-2 [16].

Traditionally, the immunoassay and the molecular assay require labor-intensive procedures to operate instruments in centralized laboratories. In recent years, due to the characteristics of accurate fluid manipulation and low sample volume consumption, microfluidics technology with a pump or capillary force along microchannels has made biomedical testing simpler and more accessible to the POC settings [17,18,19,20,21]. Alternatively, digital microfluidics (DMF), requiring no sophisticated mechanical structures, handles droplet-based bioassays with electrowetting-on-dielectric (EWOD) [22,23]. This approach provides great potential to obtain diagnostic results from samples with small volumes in a programmable way. Moreover, for the simple parallel-plate configuration, the DMF chips are ready to integrate various detection techniques [24,25] as a convenient diagnosis platform.

Without closed microchannels, DMF chips can be massively produced by printed circuit board (PCB) and injection molding processes without microfabrication procedures [26]. Several commercial DMF products have been developed for diagnostic assays, including NAATs, immunoassays, and enzymatic assays [27]. For example, Advanced Liquid Logic, Inc., demonstrated magnetic-bead-based immunoassays to detect cardiac troponin I from whole blood samples [28,29] and on-chip DNA amplification of methicillin-resistant *Staphylococcus aureus* (MRSA) using qPCR on DMF cartridges [28]. Illumina, Inc., developed the NeoPrep library preparation system with a replaceable 16-sample NeoPrep library chip by adopting DMF for the sample preparation workflow for DNA and RNA sequencing, saving hands-on time and reducing the chances for human error [30]. GenMark Diagnostics, Inc., launched the ePlex system with EWOD-based chips for the detection of DNA and RNA targets [31]. Recently, the ePlex Respiratory Pathogen Panel 2 from GenMark Diagnostics is used to detect variant SARS-CoV-2 strains by targeting two unique regions of the N gene [32] and granted an emergency used authorization (EUA) by the U.S. Food & Drug Administration (FDA). Baebies, Inc., launched SEEKER and FINDER, two DMF-based diagnosis systems for blood testing in newborn diseases screening [33,34]. In addition, they announced that the FDA acknowledged emergency used notification (EUN) for an RT-qPCR test to detect SARS-CoV-2 on the FINDER 1.5 instrument [35]. The demonstrated DMF technology greatly reduces sample and reagent consumption and manual operations, which makes it advantageous to conduct replicate assays simultaneously on the same chip.

Here, we presented a DMF cartridge that is driven and accessed by electric, thermal, and optical modules on the system to detect the SARS-CoV-2 N gene by qPCR with two sets of primer and probe, N1 and N2, based on the diagnostic panel from the U.S. CDC [16]. The functions and characteristics of the DMF system and PCB-based cartridge were verified to ensure proper qPCR amplification procedures. In addition, the N gene of SARS-CoV-2 was detected with qPCR; results demonstrated the feasibility of our DMF system in NAATs.

## 2. Materials and Methods

### 2.1. Reagents and Chemicals

The N gene cDNA template (SARS-CoV-2 RUO plasmid controls) of SARS-CoV-2 with a stock concentration of 200,000 copies/μL was purchased from Integrated DNA Technologies (IDT, Coralville, IA, USA). The qPCR primer-probe mix for N1 and N2 referring to the U.S. CDC were from IDT. The TaqMan^TM^ Fast Advanced Master Mix (2X) for two-step qPCR in gene expression and quantitative analysis produced by Applied Biosystems^TM^ was from Thermo Fisher Scientific (Waltham, MA, USA). For optical calibration, the green fluorescent dye, fluorescein (excitation/emission, 494 nm/512 nm), was from Fluka (Buchs, Switzerland). The surfactant, Tween 20 from Sigma-Aldrich (St. Louis, MO, USA), was supplemented to prepare all solutions to reduce the surface tension and biofouling of the droplets and thus to improve EWOD. The environmental fluid, hexadecane (99%, Thermo Fisher Scientific), was used to reduce friction and prevent aqueous droplets from evaporating.

### 2.2. Sample Preparation

Fluorescein solutions with varying concentrations from 100 nM to 800 nM were prepared to calibrate the optical module and evaluate the linearity of the fluorescence signal. Fluorescein was added to the sodium hydroxide (Thermo Fisher Scientific) solution and diluted with 2-fold serial dilutions. For qPCR testing of the SARS-CoV-2 N gene, we prepared a 20 μL reaction mix that contained 1 μL cDNA template of varied concentration, 1.5 μL primer-probe mix, 10 μL of DNA master mix, and 7.5 μL nuclease-free water. Tween-20 with a final concentration of 0.1% was used in aqueous reagents. Ten-fold serial dilutions of the stock cDNA template of SARS-CoV-2 N gene (200,000 copies/μL) were conducted to prepare varied template concentrations (20,000, 2000, 200, and 20 copies/μL). Subsequently, cDNA template samples with various concentrations (volume 1 μL each) were used to prepare 20 μL reaction mix with final concentrations of 10,000, 1000, 100, 10, and 1 copy/μL. For on-chip qPCR procedure of SARS-CoV-2 N gene, each concentration was duplicated four times.

### 2.3. Design and Fabrication of DMF Cartridge

**Figure 1:**
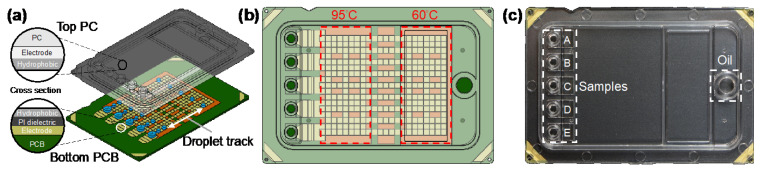
Digital microfluidic (DMF) cartridge for quantitative polymerase chain reaction of severe acute respiratory syndrome coronavirus 2 (qPCR SARS-CoV-2) testing. (**a**) Schematic illustration of the cartridge composed of top polycarbonate (PC) and bottom printed circuit board (PCB) plates with multiple droplet tracks. (**b**) The design of the driving and reservoir electrodes and two temperature zones designed to perform qPCR in the cartridge. (**c**) The assembled and packaged cartridge showing five inlets and reservoirs for samples and an inlet for oil.

The designed DMF qPCR cartridge (6 cm × 4 cm) consisted of a transparent top plate made of injection molding of polycarbonate (PC) and a bottom plate fabricated with PCB processes, as shown in Figure 1a. The inner surface of the PC top plate was coated with a conductive polymer (thickness < 50 nm) and fluorinated hydrophobic (thickness < 300 nm) layers. The opposing inner surface of the PCB bottom plate containing patterned copper electrodes (thickness ~30 μm) was covered by a polyimide (PI) dielectric film (thickness 38 μm) and a fluorinated hydrophobic layer (thickness < 300 nm). The top and bottom plates were electrically connected via a conductive gasket and then sealed with glue, forming a sandwich structure. The cartridge materials and fabrication were provided by GEMFluidix’s proprietary services. The PCB bottom plate accommodated totally 274 square driving electrodes (pitch 1.5 mm) for programmable droplet movements; the parallel electrode routes offered multiple droplet tracks between temperature zones (Figure 1b) for concurrent multiple qPCR reactions. The PCB was also designed to have five sets of reservoir electrodes corresponding to the inlets and reservoirs on the top plate (Figure 1c). The PC top plate provided electric ground and mechanical structures of five independent inlets and reservoirs (A–E) for varied samples in qPCR reaction mixes as well as an oil inlet for the environmental fluid. With the 1.5 mm pitch of square driving electrodes and the 340 μm gap height between top and bottom plates, the volume of a droplet generated from a reservoir by a single driving electrode was approximately 0.765 μL.

### 2.4. Experimental Setup

The DMF cartridge was controlled and accessed with electrical, thermal, and optical modules. The electric module drove droplets on the DMF cartridge with driving signals from direct current (DC) to above 1 kHz alternating current (AC) with the maximum voltage of 300 V. The thermal module consisted of PI heating films (Taiwan KLC, Taichung City, Taiwan) and precision epoxy thermistors (Adafruit, New York, NY, USA) for feedback to meet temperature requirements of qPCR amplification. The optical module was composed of a camera (Teledyne FLIR, Wilsonville, OR, USA), a fluorescence filter cube for FAM fluorophore (excitation/emission: 495 nm/520 nm), and a LED light source (Lumileds, San Jose, CA, USA) to obtain the fluorescence signal of each cycle during the qPCR reaction.

### 2.5. Performance of DMF System

The DMF cartridge was evaluated for droplet volume consistency, temperature uniformity, and fluorescence intensity linearity. First, the electric module and fluidic performance were tested based on the droplet volume consistency. Repeatable droplet generation with precise droplet volume is important to assay quantification in determining the copy-number concentration [36,37]. Three droplets were generated from each track/reservoir. The top-view area of the generated droplets was measured using ImageJ software (v. 1.51j8, National Institutes of Health, Bethesda, MD, USA) [38] then converted to volume by multiplying with the droplet height. Second, the thermal module was examined. The cartridge temperature, controlled by heating films and feedback thermistors, is crucial to qPCR reaction. The targeted temperature was calibrated and adjusted using thermocouples (Omega Engineering, Norwalk, CT, USA) directly inserted into the oil (i.e., hexadecane) environment in two temperature zones of the DMF cartridge, and each data point was replicated three times. The temperature uniformity was also studied with an infrared thermal imager (A6700sc, Teledyne FLIR). Third, the optical module was assessed based on the linearity of the fluorescence intensity of droplets having fluorescein dye at varied concentrations.

### 2.6. On-Chip qPCR Procedure of SARS-CoV-2 N Gene

Based on the suggested U.S. CDC diagnostic panel [16], our DMF cartridge was designed to detect the N gene of the SARS-CoV-2 virus with two sets of primer and probe for N1 and N2 target regions through qPCR amplification. Using TaqMan^TM^ Fast Advanced Master Mix, a set of temperature and cycle duration was adopted for qPCR reactions: 1 cycle of enzyme activation at 95 °C for 2 min, followed by 40 cycles of a thermal profile of 1 s at 95 °C for denaturation, and 20 s at 60 °C for annealing and extension. The droplets containing specific forward and reverse primers and probes for qPCR reaction were generated from different reservoirs. The droplets were driven between the 95 °C and 60 °C regions for thermal cycling. With the current cartridge design and droplet speed, a temperature ramp rate of 1.75 °C/s was adopted to complete single “up ramp” or “down ramp” in 20 s. The thermal profile and temperature ramp rate took around 42 min to complete 40 cycles of qPCR. It is noteworthy that the ramp rate is easily programmable by adjusting the droplet speed; the distance between temperature zones can also be redesigned.

### 2.7. Data Analysis

After each thermal cycle, the fluorescence emission intensity of all droplets on varied tracks was obtained by analyzing the captured image using ImageJ software. The baseline and the threshold signal level were determined by LinRegPCR (2020.0, Dept. Medical Biology Amsterdam UMC, Amsterdam, The Netherlands) [39] that analyzed raw data of fluorescence emission intensity and estimated the exponential phase by setting the window-of-linearity. The delta fluorescence intensity (ΔFI) was obtained by subtracting the baseline intensity from the captured intensity along the progression of the qPCR reaction. By expressing ΔFI on the logarithmic scale, the threshold cycle (Ct) was determined for quantification analysis. Moreover, the amplification efficiency of the qPCR reaction was evaluated by performing a dilution series experiment. The slope of the standard curve between Ct and the logarithm of the concentration of template was translated into efficiency by:

$$\mathrm{Efficiency}=10^{\left(-\frac{1}{\mathrm{slope}}\right)}-1$$

The qPCR efficiency should be between 90–110%, and 100% denotes the most optimized efficiency in qPCR amplification [40,41].

## 3. Results

### 3.1. Droplet Volume Consistency

**Figure 2:**
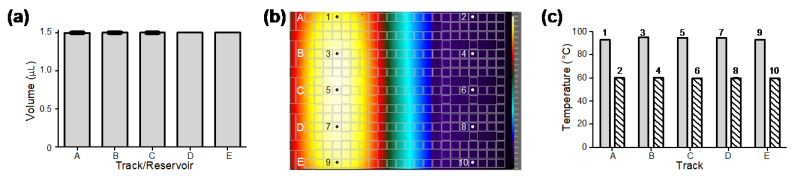
Performance tests of droplet volume consistency and temperature uniformity of the DMF cartridge. (**a**) Consistent volume of droplets generated on different droplet tracks from their corresponding reservoirs (*n* = 3 for each track/reservoir). (**b**) Infrared thermal imaging of a DMF cartridge filled with hexadecane providing uniform temperature distribution in two temperature zones for qPCR amplification. (**c**) Direct thermocouple measurements of hexadecane at locations 1–10 shown in (**b**) cross droplet tracks in 95 °C and 60 °C temperature zones (*n* = 3 for each location).

The electric module and fluidic performance were tested based on the droplet volume consistency. The volume of droplets on tracks A–E generated from reservoirs A–E (seen in Figure 1c) was obtained by image analysis. Three droplets were generated from each track/reservoir. As the results show in Figure 2a, the average volume of the droplets generated by two driving electrodes was approximately 1.5 μL. The coefficients of variation (CV) in droplet volume from different reservoirs and droplet tracks were less than 0.15%, which indicated that the generated droplets were highly consistent. Droplet generation and movement can be seen in the Supplementary Materials, Video S1.

### 3.2. Temperature Uniformity

To evaluate the stability of the thermal module and the uniformity of the cartridge, we measured the temperature with an infrared thermal camera and thermocouples. First, the cartridges filled with hexadecane were placed above the thermal module and observed under the infrared camera. As can be seen in Figure 2b, the infrared thermal image showed uniform temperature distribution in each temperature zone. The temperature uniformity was further confirmed by direct measurements with thermocouples that were inserted into hexadecane in the cartridge at multiple locations 1–10 (Figure 2b) on tracks A–E in two temperature zones. As shown in Figure 2c, the temperature of hexadecane in the two temperature zones measured 94 ± 1 °C and 60 ± 0.5 °C on different droplet tracks. The results demonstrated high accuracy and stability of temperature at different locations on both temperature zones.

### 3.3. Fluorescence Intensity Linearity

**Figure 3:**
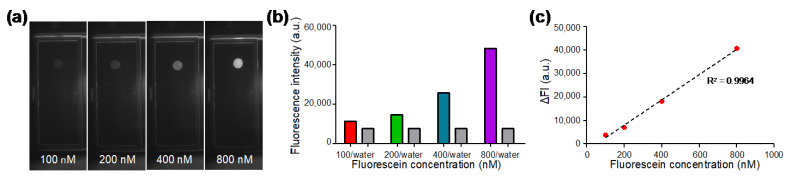
Characterization of the optical module with serially diluted fluorescein solutions. (**a**) Fluorescence images of fluorescein solution droplets at different concentrations captured by the optical module. (**b**) Fluorescence intensity of water droplets and fluorescent droplets at various concentrations. (**c**) Standard calibration curve for fluorescein concentration between 100 nM and 800 nM showing desirable linearity for qPCR.

Fluorescent droplets containing fluorescein with concentrations of 100, 200, 400, and 800 nM were generated on the DMF chip and the fluorescence images (Figure 3a) were captured by the optical module consisting of a camera, a fluorescence filter cube, and a LED. In each experiment, a water droplet without dye was also generated as a control. As shown in Figure 3b, the fluorescence intensity of dyed droplets and undyed water droplets were analyzed from the capture images. The fluorescence intensity of water droplets was between 7500 to 7600 a.u.; whereas the fluorescence intensity of the dyed droplets increased with the concentration of fluorescein. As shown in Figure 3c, in order to evaluate the linearity of our optical system, the delta fluorescence intensity (ΔFI) was plotted against varied fluorescein concentration, where ΔFI was obtained by subtracting the value of water from the value of fluorescein. The standard calibration curve showed high linearity with the coefficient of determination R^2^ being 0.9964, indicating that desirable fluorescence linearity can be obtained by our optical module for qPCR analysis.

### 3.4. qPCR Amplification of N1 and N2 Target Regions of SARS-CoV-2 N Gene on DMF Cartridge

**Figure 4:**
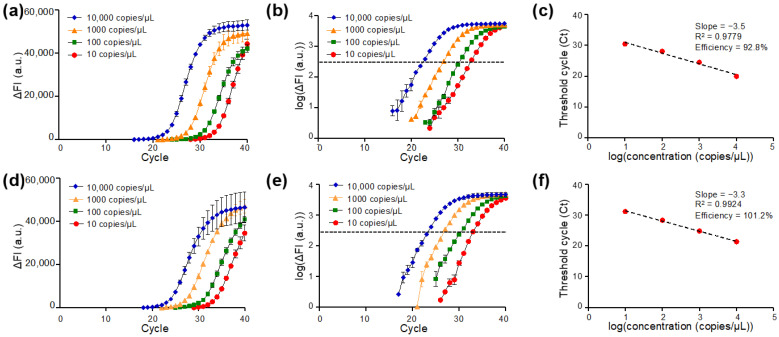
qPCR of the SARS-CoV-2 N gene with U.S. Centers for Disease Control and Prevention (CDC)-referenced N1 or N2 primer-probe set with 1.5 μL droplets on the DMF cartridge: (**a**–**c**) N gene detected by N1 primer-probe set and (**d**–**f**) by N2 primer-probe set. qPCR amplification curves of delta fluorescence intensity (ΔFI) against cycle number for various template concentrations detected by (**a**) N1 and (**d**) N2 primer-probe sets. Logarithmic plots of ΔFI against cycle number for various concentrations of reactions carried out by (**b**) N1 and (**e**) N2 primer-probe sets. Standard curve and qPCR efficiency of (**c**) N1 and (**f**) N2 primer-probe sets targeting to SARS-CoV-2 N genes on the DMF cartridge (*n* = 4 for each concentration).

The amplification plots of qPCR targeting N1 (Figure 4a–c) and N2 (Figure 4d–f) regions of the SARS-CoV-2 N gene are presented. Typical amplification curves for the N1 target region in qPCR on DMF cartridges were observed, which are similar to standard qPCR reactions in Eppendorf tubes with benchtop qPCR instruments [42]. As the amplification proceeded, the higher concentration of the cDNA template corresponded to an earlier rise in the ΔFI curve. The results showed that the DMF cartridge was able to detect N gene cDNA with a concentration of 10 copies/μL, which corresponded to 15 copies of cDNA in the manipulated droplets (volume 1.5 μL) with the N1 primer-probe set. When ΔFI was plotted in the logarithmic scale for four samples with different N gene cDNA concentrations, the cycle number of each sample concentration corresponding to the same threshold signal level was determined as the Ct value (Figure 4b). The Ct values were plotted against the logarithm of the N gene cDNA concentration for evaluating the performance of the DMF cartridge, as illustrated in Figure 4c. Linear regression of the standard curve shows the slope of −3.5 and the coefficient of determination R^2^ of 0.9779. The qPCR efficiency was calculated as 92.8%, which is in the range of acceptable values.

The same analyses were applied to study qPCR of the N gene detected by N2 amplicon. The ΔFI curve is shown in Figure 4d. The logarithmic plot of ΔFI versus qPCR cycle number revealed similar Ct values when the same sample was tested by N2 (Figure 4e) and N1 (Figure 4b), suggesting the test stability on the DMF cartridges. The Ct values were used to plot the standard curve for quantification analysis as shown in Figure 4f. A linearity of the standard curve was observed with the slope of −3.3 and the R^2^ of 0.9924. A fairly high qPCR efficiency (101.2%) was obtained. The results from N1 and N2 gene targets demonstrated that the DMF cartridge is with great potential for gene quantification.

## 4. Discussion

As one of the advanced microfluidic techniques, DMF demonstrates many advantages due to its portability and capacity for automation and real-time monitoring. First, controlled by the electric module, our DMF cartridge precisely manipulated droplets for qPCR with volume of 1.5 μL that is more than 13 times smaller than the conventional qPCR assays (20 μL for benchtop qPCR machines). Second, with the thermal module, the demonstrated DMF qPCR cartridge set up precise temperature zones. Because the thermal cycle was determined by droplet motion, temperature overshooting [43] was avoided and the temperature and ramp rate could be controlled for better thermal cycling. For example, on the same DMF cartridge, we modified the thermal profile by increasing the temperature ramp rate to 5 °C/s (droplet speed 3 mm/s) and decreasing the annealing duration to 10 s (60 °C, 10 s; 95 °C, 1 s; ramp 7 s) to successfully amplify the template with 40 cycles of PCR in 17 min. Third, the optical module automatically recorded the fluorescence after each cycle, which enabled real-time analysis and simplified the entire workflow from sample to result.

Compared with other commercial DMF-based SARS-CoV-2 tests [32,35], our demonstrated cartridge provided both multiplex and real-time tests based on the U.S. CDC diagnostic panel. It held five inlets and reservoirs that could handle both N1 and N2 target regions of the N gene, as well as a human RNase P gene (RP) control, a positive control, and a no template control (NTC) altogether on the same DMF qPCR cartridge. Thus, more target genes or other pathogens could be tested by redesigning the cartridge and assay. However, our cartridge did not offer sample-to-answer functions as the commercial DMF-based SARS-CoV-2 tests do. The current off-chip sample preparation steps including bead-based extraction and reverse transcription could also be integrated on our cartridge in the future [44,45].

With high qPCR efficiency on our DMF cartridge, we were able to detect 10 copies/µL N gene targets in 1.5 μL droplets, or with the initial concentration of 15 copies/droplet. With the current 42 min thermal profile (60 °C, 20 s; 95 °C, 1 s; ramp 20 s), 1 copy/µL (1.5 copies/droplet) samples were not consistently detected on our cartridge because the probability of an empty droplet was high (estimated 22% from Poisson distribution). The limit of detection (LOD) between 1 copy/µL and 10 copies/µL will be carefully verified in the future. The on-chip qPCR can be improved by modulating the experimental parameters and redesigning the cartridge. Specifically, we can optimize the annealing temperature and duration or concentration of master mix to increase the amplification efficiency and sensitivity as well as to reduce the reaction time [46]. The 17 min thermal profile (60 °C, 10 s; 95 °C, 1 s; ramp 7 s) demonstrated the beginning of the optimization. Finally, the droplet volume is adjustable by the dimension of the driving electrodes, and the distance between temperature zones can be modified by redesigning the cartridge.

## 5. Conclusions

The DMF qPCR cartridge was designed and tested to detect the N gene targets of SARS-CoV-2 with the U.S. CDC-referenced N1 and N2 primer-probe sets. With reduced sample consumption, the amplification performance of the DMF cartridge was comparable with that of benchtop qPCR instruments. The DMF cartridge showed reliable droplet generation with consistent volume, uniform temperature control, and desirable fluorescence readout, all contributing to the implementation of qPCR POC testing.

## Supplementary Materials

The following supporting information can be downloaded at: https://www.mdpi.com/article/10.3390/mi13020196/s1, Video S1: Demonstration of droplet movement on the DMF cartridge played at 3× speed.
